# A Benford's law based method for fraud detection using R Library

**DOI:** 10.1016/j.mex.2021.101575

**Published:** 2021-11-11

**Authors:** Caio da Silva Azevedo, Rodrigo Franco Gonçalves, Vagner Luiz Gava, Mauro de Mesquita Spinola

**Affiliations:** a University of São Paulo, Brazil; b Universidade Paulista, Brazil; c Instituto de Pesquisas Tecnologicas, Brazil

**Keywords:** Benford's law, Statistical antifraud analysis, Anomaly detection, Bolsa familia, Social welfare programs

## Abstract

Benford Law (BL) states that the occurrence of significant digits in many natural and human phenomena data sets are not uniformly scattered, as one could naively expect, but follow a logarithmic-type distribution. Here, we present a method that consists of the use of BL analysis over first and first-two digits, three statistical conformity tests – Z-statistics, Mean Absolute Deviation (MAD) and Chi-square (χ2) as well as the summation test which looks for excessively large numbers, having fraud detection as one of its application. We developed the method for fraud detection in the case of the Brazilian Bolsa Familia welfare program. In this case, we submitted four periods of Brazilian welfare program payments to the method with a dataset of 13,442,529 records. We provide a practical implementation of the method based on open-source R library released on a public repository. Furthermore, code implementation of the algorithm as well as datasets are freely available. Advantages of the algorithm are listed below:

• The method was developed based on open source libraries

• The technique is simple, rapid and ease of use

• Easily applicable to other social welfare program auditing

## Graphical abstract

**Figure:**
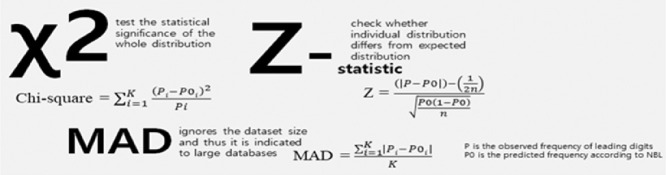

Specification table

**Table:** 

Subject Area:	Computer Science, Data Analytics, Statistics
More specific subject area:	Benford's law, Statistical antifraud analysis, Anomaly detection
Method name:	BL-based method for fraud detection using R
Name and reference of Original method:	NA
Resources availability:	R-script and data set files https://github.com/cazevedo1977/brazilian-welfareprogram-bolsafamilia-dataanalysis https://www.kaggle.com/caioazevedo/brazilian-welfareprogram-bolsafamilia-dataanalysis

## Method details

### Background

Benford's Law (BL) [2] states that smaller digits occur more frequently than larger digits in natural phenomena, contradicting the common sense that all digits occur with the same frequency in a uniform distribution. Thus, in a set of numerical data or anomalous numbers as postulated by Benford [2], the number 1 would appear as the most significant digit about 30% of the time, while digit 9 would appear less than 5% of the time.

Nigrini's works [5], [6], [7] propagated among scholars and practitioners that BL can be used as a forensic accounting and auditing tool for financial data and since then it has been used as a sophisticated statistical technique in fraud detection process, such as funds distribution among people such as governmental countrywide welfare programs.

This paper accompanies the paper entitled “A Benford's Law based methodology for fraud detection in social welfare programs: Bolsa Familia analysis” Azevedo *et al*. [1], which presents a systematic method based on BL analysis over first and first-two digits, as well as three statistical conformity validation tests and the summation test, having fraud detection as one of its application.

As case study, the accompanying research paper explains how BL-based proposed method can be used to ensure that monthly beneficiaries withdrawals values fit BL, aiming to support auditors being accurate on selecting regions suspected of frauds. The details related to the fraud detection method are reported in this paper.

Method and Data Sets

BL states a logarithmic law of frequency of leading digits. This law is defined as follows:

(1) P(d)=log10(1+1/d),d=1,2,…,9

where P is the probability of leading digit (d) occurrence.

This law was expanded by Hill [9] that postulated that the law is valid not only for the first digit but also to the others.

(2) $$P\left(D_{1}=d_{1,\ldots ,}D_{k}=d_{k}\right)=log_{10}\left(1+\;\frac{1}{\sum_{i=1}^{k}10^{k-i}d_{i}}\;\right)$$

where d1 ∈ {1, …, 9}, and all other dj ∈ {0,…,9}, j=2,…,k.

That means that a randomly selected natural number should begin with the digit 1 in about 30% of the time: more precisely, the proportion should be 0.301. The frequency of numbers with leading digit 2 should be about 18% (obtained from log10(3/2)), those with leading digit 3 should be about 12% (from log10(4/3)), and so on until the frequency of 8’s should be 5.1% and that of 9’s should be 4.6%.

In order to check whether or not, significant digits values conform to Benford's law, we applied Chi-square (χ2), Z-statistic and Mean Absolute Deviation (MAD) as goodness-of-fit tests to validate observed frequency of leading digits on sample datasets (P) against predicted frequency according to BL (P0). Moreover, summation test is used to identify numbers that are large compared to the norm for that data.

#### Statistical Significance level

Every statistical analysis lay down whether results have statistical significance according to pre-established limits. Significance levels show how likely a pattern in your data is due to chance. The significance level, also denoted as alpha or α, is the probability of rejecting the null hypothesis when it is true. The most common level used to mean something is good enough to be believed is 95%. Thus, a significance level of 95% indicates a 5% risk of concluding that a difference exists when there is no actual difference.

#### Statistical tests

The proposed methodology uses three statistics tests – Chi-square (χ2), Z-statistic and Mean Absolute Deviation (MAD) for examining the statistical significance. First, Z-statistic check whether the individual distribution significantly differs from Benford's Law distribution. Here, individual distribution refers to first digit and first-two digits. Mathematically, the Z-statistic considers the absolute magnitude of the difference (the numeric distance from the actual to the expected), the size of the data set, and the expected proportion. The formula adapted from Fleiss [4] is shown in equation 3.

(3) $$Z=\;\frac{\left(\left|p-p_{0}\right|\right)-\left(\frac{1}{2n}\right)}{\sqrt{\frac{p_{0\left(1-p_{0}\right)}}{n}}}$$

Where P is the observed frequency of leading digits.

P_0_ is the expected frequency under Benford´s law.

*n* is the number of records.

The term $\left(\frac{1}{2n}\right)$ is a continuity correction term and should be used only when it is smaller than (|p-p_0_|).

If the values of Z-statistic exceed the critical value 1.96, the null hypothesis (H_0A_) is rejected at 5% of significance level, the most usual level as proposed by Nigrini [7]. It is also possible to adopt critical values of 2.57 and 1.64 to 1% and 10% significance levels, respectively.

Let us consider a significance level of 5 percent. This means that we should not be too concerned if we have four or five (90 possible first-two digits 5 percent) significant spikes for any data set [7].

Second, chi-square is used to test the statistical significance to the whole distribution in observed frequency of first digit and first two digits against their expected frequency under Benford's Law. The null hypothesis (H_0A_) is that the digits conform to Benford's Law. The chi-square statistic is calculated as is shown in equation 4.

(4) $$Chi-square=\;\sum_{i=1}^{K}\frac{{\left(Pi-P0i\right)}^{2}}{Pi}$$

Where P and P0 represent the actual count and expected count respectively, and K represents the number of bins (90, because of there are 90 first-two digits). The number of degrees of freedom is K–1, which means that for the first-two digits, the test is evaluated using 89 degrees of freedom. A calculated chi-square is compared to a critical value and is as higher it is, the more data deviates from Benford's Law.

As well as Z-statistic, Chi-square also uses 5% of significance level as Nigrini [7] recommendation for Benford's Law validation, in this case 15.507 and 112.022 as critical values for first and first-two digits tests, respectively. Once they exceed, the null hypothesis (H_0A_) is rejected.

In this work, just like Z-statistics, it is assumed 5%, although chi-square limits may also consider 1%, 5% and 10% as detailed on [1], [3], [4], [6], [7].

Critical Values of the Chi-Square Distribution are freely available on Engineering Statistics Handbook provided by [8].

Lastly, Mean Absolute Deviation (MAD) test. This test ignores the dataset size and thus it is indicated by [7] to large databases. It is mathematically expressed as:

(5) $$\text{MAD}\;=\;\frac{\sum_{i=1}^{K}\left|P_{i}-P0_{i}\right|}{K}$$

Where K means the number of bins (which equals 90 for the first-two digits), P represents the actual proportion and P_0_ the expected proportion under BL.

The absolute symbol means that the deviation is given a positive sign irrespective of whether it is positive or negative. The absolute deviations need to be added together and divided by the number of bins, i.e. the average (or mean) absolute deviation.

However, there are no objective critical scores for MAD test, Nigrini [7] proposed critical scores for nonconformity, for conformity, and for some in-between categories based on personal experience with everyday data tables that were tested against Benford's Law as detailed in Table 1. This table gives the critical values for the first and first-two digits tests.

**Table 1:** Cut-off scores and conclusions for calculated MAD values.

First Digits		First-Two Digits	
0.000 to 0.006	Close conformity	0.000 to 0.012	Close conformity
0.006 to 0.012	Acceptable conformity	0.012 to 0.018	Acceptable conformity
0.012 to 0.015	Marginally acceptable conformity	0.018 to 0.022	Marginally acceptable conformity
above 0.015	Nonconformity	above 0.022	Nonconformity

**Source:** (adapted from [7]).

**Fig. 1:**
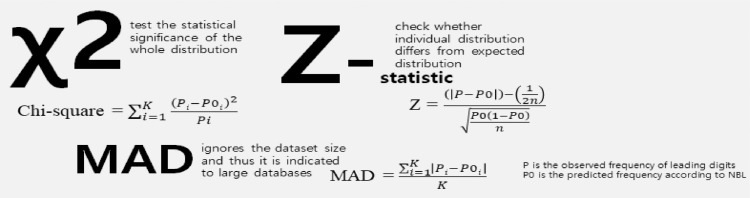
Proposed goodness-of-fit statistical tests

Fig. 1 below illustrates BL conformity statistical tests, proposed in this work.

#### Summation test

Classified by [7] as one of the advanced Benford's Law tests, as well as the second-order test, the summation test looks for excessively large numbers in a dataset. It identifies numbers that are large compared to the norm for that data. The test was proposed by Nigrini [7] and it is based on the fact that the sums of all numbers in a Benford distribution with first-two digits (10, 11, 12, …99) should be the same. Therefore, each of the 90 first-two digits groups sum proportions should be equal, i.e. 1/90 or 0.011, and spikes indicate that there are some large single numbers or set of numbers. To the scope of this work, let us assume 25% of upper tolerance and thus the proportional critical value is 0.01375.

Nevertheless, Nigrini [7] verified that summation theorem has shown that real-world data sets seldom show the neat, once they have abnormal duplications of large numbers. In general, it is not possible to say whether the summation spikes are caused by a handful (one, two, or three) of noticeably big numbers or an abnormal duplication of a few hundred moderately big numbers without a closer look at the data.

Data Samples: Bolsa Família Program

The data used in this study is publicly available on Federal Government “Portal da Transparência” regarding January and March of 2018 and 2019. In 2004, the Brazilian Government instituted the Bolsa Familia as a social assistance program. Bolsa Familia is a welfare program which is the flagship of social protection to the poor by providing financial aid to destitute families. By the time of our research, it reached approximately 13.4 million households monthly, corresponding to the poorest population of Brazil. Its primary goals are to fight hunger and poverty; strengthen access to the public service network, especially to education, health, and social assistance; promote intersectionality integration and public policy synergy; and encourage sustained empowerment of beneficiary families (Brazil 2004).^1^

The program consists of conditional cash transfer being given preferentially to females in exchange for children attending school and receiving regular medical check-ups in public clinics. This work used withdrawal values, as it consists of real benefits amount monthly paid to the citizens.

All data files were downloaded on May 10th, 2020 and they are available at http://www.portaltransparencia.gov.br/download-de-dados/bolsa-familia-saques, by choosing the year and month and clicking the button “Baixar” (download). File format is csv and they are ready to be analyzed by R scripts.

It is important to highlight that we notice that data have been changed over the time due to accounting adjustments and thus we provide datasets exactly as used in this work at: https://www.kaggle.com/caioazevedo/brazilian-welfareprogram-bolsafamilia-dataanalysis.

The data set dictionary is detailed on Table 2 below:

**Table 2:** Withdrawal data file dictionary

Column	Description
Reference year/month	Payroll year/month
Accounting period year/month	Year/month of the payment
UF	program beneficiary federation unit initials
Municipality SIAFI code	program beneficiary municipality SIAFI code. SIAFI (Financial Administrative Integrated System)
Municipality Name	program beneficiary municipality name
Beneficiary NIS	program beneficiary NIS. Created by Caixa Econômica Federal, NIS means a social identification number provided to Brazilian citizens when they join any social program, i.e., Bolsa Familia, FGTS, working papers, became INSS taxpayer or started labouring life for private or public companies. (Source: Caixa Econômica Federal)
Beneficiary Name	Bolsa família program beneficiary name
Withdrawal date	Date in which the withdrawal was realized
Withdrawal value	Beneficiary payment value in BRL

**Source:** (adapted from Brazilian transparency portal, 2019. Available at http://www.portaltransparencia.gov.br/pagina-interna/603401-dicionario-de-dados-bolsa-familia-saques)

**Fig. 2:**
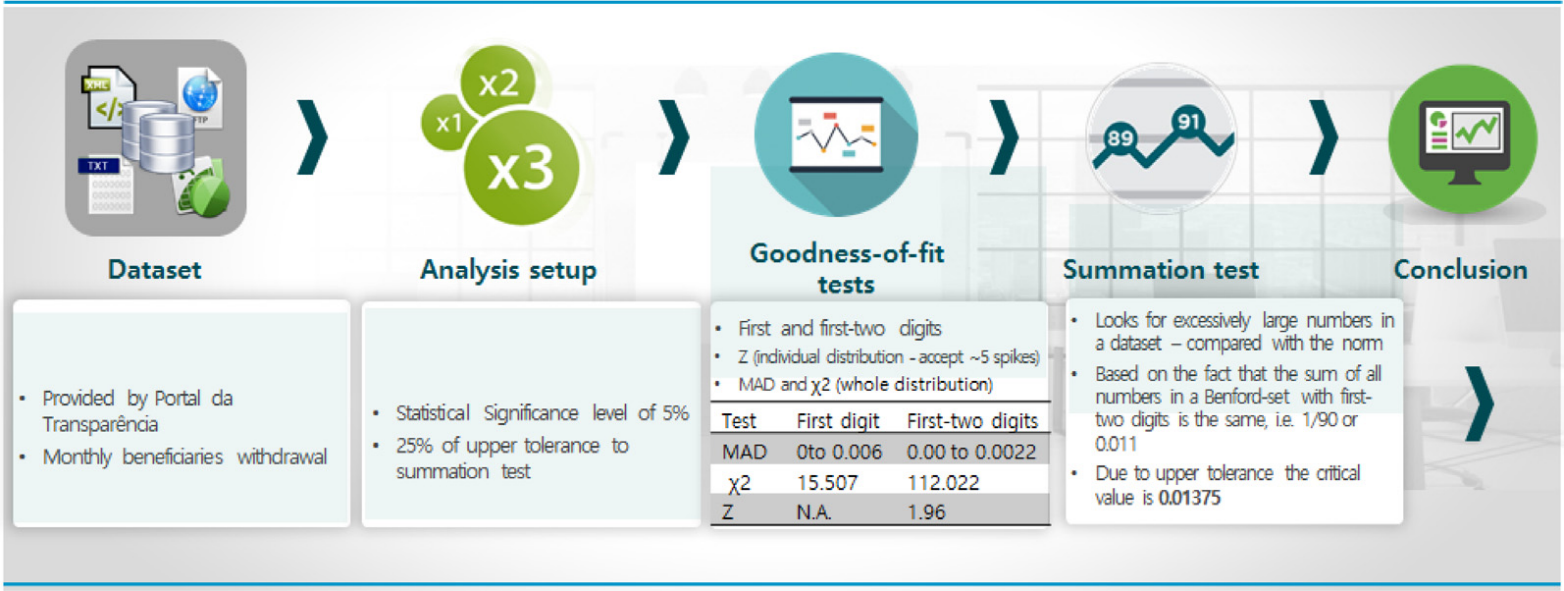
A diagram illustrating the steps of the method

In overview, as shown in Fig. 2, the BL-based methodology proceeds as follows:

• First, data set is collected from suggested repository
• Significance level and range of tolerance definitions
• Data set is submitted to goodness-of-fit tests
• Data set is submitted to summation test

## Material

To perform the analysis, two R scripts were programmed to read and categorize the information contained in the data files. R scripts also contains necessary routines to perform the analysis, check the consistence with BL distribution and summation test according to proposed method and finally produce charts and tables. All these scripts are available via GitHub in the repository: https://github.com/cazevedo1977/brazilian-welfareprogram-bolsafamilia-dataanalysis.

The scripts were developed and ready to use on RStudio IDE. RStudio is an open source integrated development environment (IDE) for R, a programming language for statistical computing. R-scripts were written having “benford.analysis” package as supporter. This library was provided by Cinelli, C. [3].

The equipment and software necessary to perform this research are:

- Notebook Lenovo intel core i7 1.8 GHz - 8th generation and 8GB RAM
- Microsoft Windows 10 Home - Version 10.0.183363
- RStudio Desktop for Windows version 1.2.1335

To use the scripts, it is important to have the dependencies properly installed in the system and to have the data sets files properly downloaded and its location in the computer properly referenced in the scripts. The scripts were programmed to work in a windows system, adaptations must be done if the user uses a different system (such as Linux or macOS). More details about the scripts see inline comments written in both files.

## Testing scenarios

On a first moment, we chose one file from Portal da Transparência regarding January 2018. As initial exploratory data analysis we submitted withdrawal values to BL tests. Once these tests failed, we changed our viewpoint and we grouped data set geographically into the Brazilian municipalities and thus submit this new data set to BL tests again.

Exploratory analysis and data preparation

As a first approach, see “Benford_BolsaFamilia_MethodsX.FirstApproach.R” R-script file. Special attention to the commands below that identify name and location of the file to be analyzed. setwd("C:/Users/cazev/OneDrive/Desktop/docs/Mestrado/paper benford law/references/datasources") dataSet <- read.csv2(file="201801_BolsaFamilia_Saques.csv",sep=';',header=TRUE,fileEncoding = "latin1")

Test variable is beneficiaries’ withdrawal values (VALOR.PARCELA attribute) andFig. 3 illustrates dataset first digit result. Orange bars represent the observed distributions and blue bars represent the Benford's law theoretical distribution. The bar chart shows that all first significative digits (FSD) distribution is different from the expected BL distribution.

**Fig. 3:**
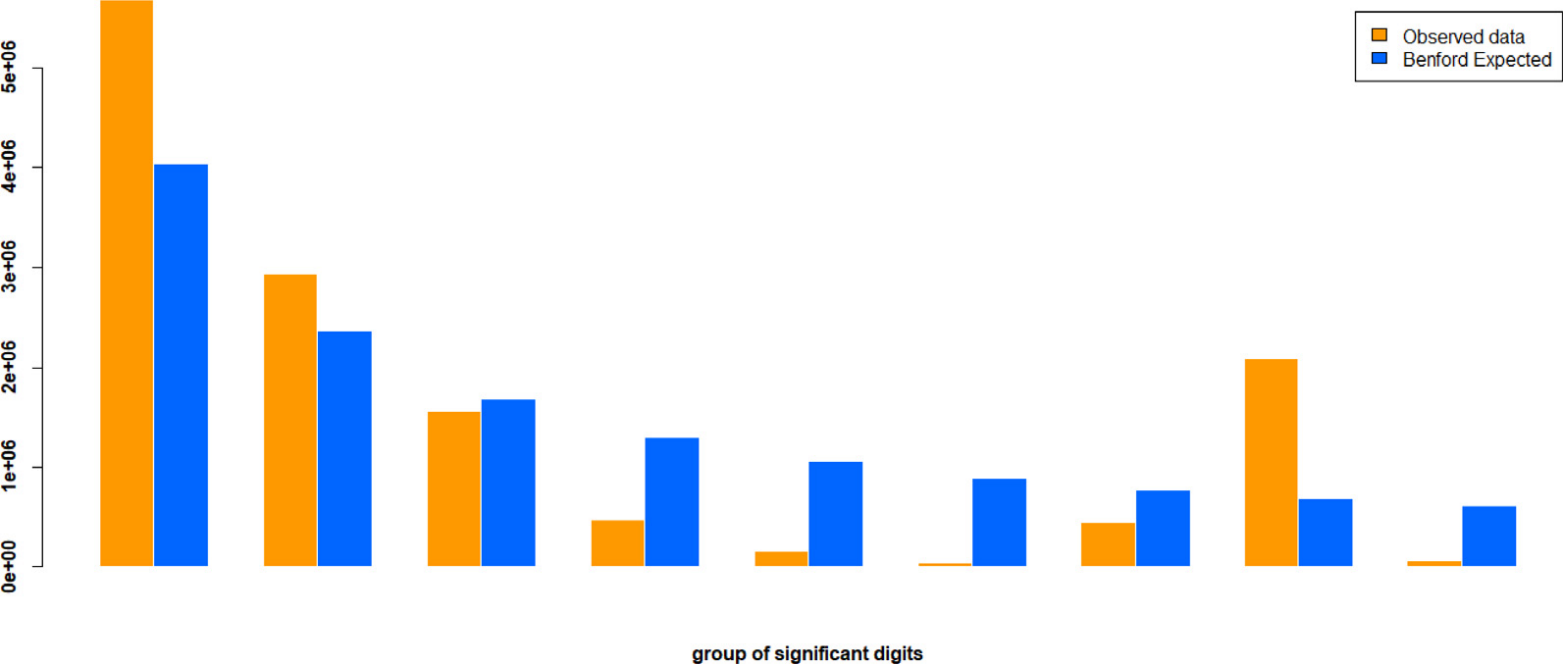
Comparison through bar charts of January 2018 withdrawal values and BL first digit occurrences. The orange bars show the actual proportions, and the blue bars show the proportions of BL.

The dataset contains 13,442,529 records and it is crystal-clear that observed and expected first significative digits distributions are completely different as reinforced by chi-square test which value is 6,480,128.958 and overwhelmingly above the critical value of 15.507, having us used 5%, of significance level. Once first-digits test failed, first-two digits tests were aborted.

Even though the first trying test failed, notice that the last line of the script is used to group original data set by Brazilian municipalities for the reasons detailed below. Take special attention to the suggested file names that must be in accordance with original files. dataSet <- myGroupDataByMunicipalities(dataSet,"BolsaFamilia_Municipalities_Jan_2018.csv")

Another perspective

A significant breakthrough has been achieved when data are geographically aggregated into the 5570 Brazilian municipalities. Thus, instead of individual withdrawal values, our test variable henceforth is the amount each municipality has spent. Doing so, our analyses run over samples of the population, i.e. the beneficiaries, which represents a natural data set as pointed out by Nigrini [7]. This sort of data aggregation may be useful in situations where test variable is spread over geographic areas.

CSV files (BolsaFamilia_Municipalities_Jan_2018.csv, BolsaFamilia_Municipalities_Mar_2018.csv, BolsaFamilia_Municipalities_Jan_2019.csv and BolsaFamilia_Municipalities_Mar_2019.csv) are available and their data dictionary is described on Table 3.

**Table 3:** Geographically aggregated data dictionary

Column	Description
Accounting period	Year/month of the payment
Municipality Name	Programme beneficiary municipality name
State	Brazilian State
Number of Beneficiaries	Total quantity of beneficiaries which payed at that municipality
Withdrawal value	Municipality accumulated payment value in BRL

**Source:** (created by authors, 2020)

Use RStudio to open and run “Benford_BolsaFamilia_MethosdsX.FinalAnalysis.R” file to proceed data analysis. Once again watch out for dataset file name and location before start code execution.

**Fig. 4:**
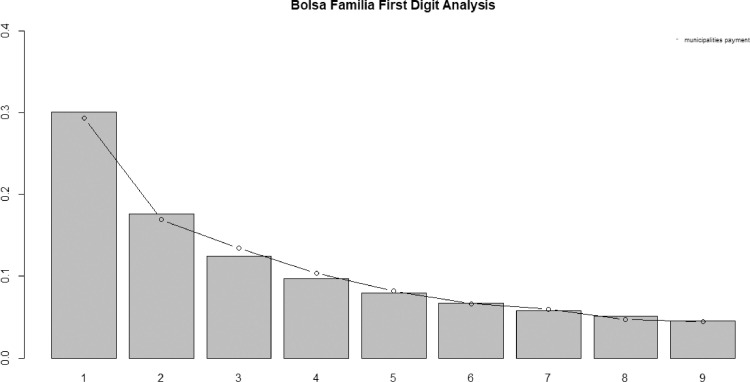
January 2018 withdrawal values and BL first digit occurrences. Bars show the actual proportions and the dots show the proportions of expected BL.

**Fig. 5:**
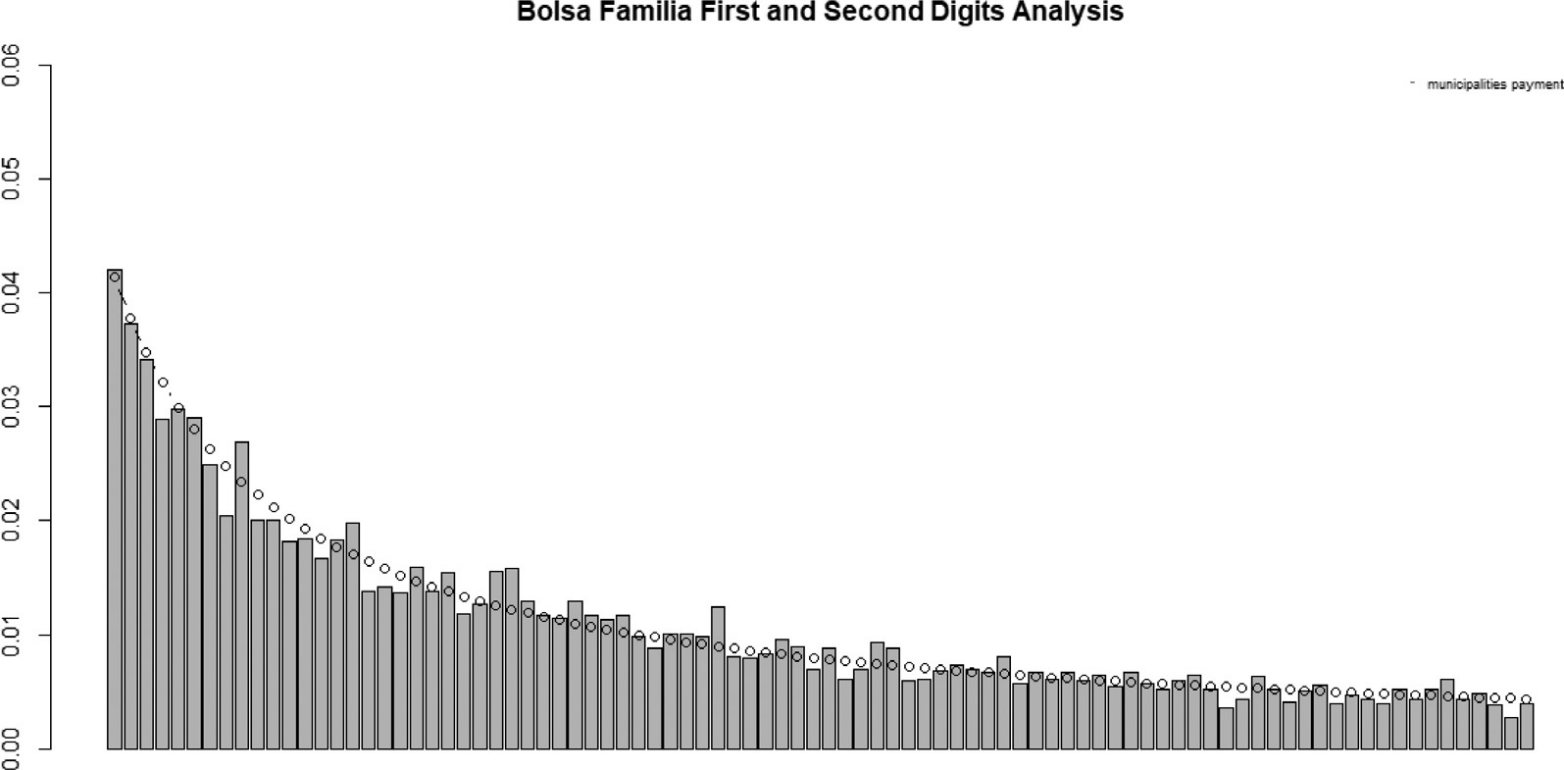
January 2018 withdrawal values and BL first-two digits occurrences. Bars show the actual proportions and dots are the expected proportions of BL.

Fig. 4, Fig. 5 illustrate data analysis from January 2018 first and first-two significative digits (FSD) frequencies respectively and the expected frequency in accordance with Benford's Law.

Proposed statistical tests corroborate bar charts visual analysis as in case of first-digit analysis chi-square of 11.932 is under critical value of 15.507. Not differently, first-two digits analysis statistical tests are also under critical values, i.e., chi-square of 80.8485 less than the critical value of 112.022 when significance level equals 5%. Moreover, there are only four Z-statistics values above the cut-off of 1.969 which are 48 (2.6486), 35 (2.3590),17 (2.0468) and 34 (1.9683) what do not clashes BL as suggested by Nigrini [7] where at 5% of significance level it is expected four or five significance spikes. Finally, MAD test scored 0.0010 far below 0.0022 limit value.

Thus, according to the methodology adopted in the scope of this work, aggregated data from January 2018 conforms BL.

Once goodness-of-fit results conforms to BL, the test variable, i.e. withdrawal values, are submitted to summation test where according to Nigrini [7], the sums of all the numbers in a Benford distribution with first-two digits 10, 11, 12, . . . , 99 should be equal, as well as their proportions, i.e. 1/90 or 0.011. The methodology of this work assumes 25% of upper tolerance and thus the proportion critical value of 0.01375.

When submitted to summation test, we select the subset from the 90 first-two digits in which the sum proportion value overcome the critical value of 0.01375. Thus, in case of Bolsa Familia benefits payment, we verify the materiality and relevance of each group of digits, in order to select those that deserve a more detailed and critical looking.

The method proposed in this paper, adapted from Nigrini [7], select top 10 highest summation test results regarding digits groups selection as shown on Table 4.

**Table 4:** Top 10 worst summation test result, number of municipalities per digits group and the total amount spent in each of the analyzed sample.

Jan 2018	Digits	Municipalities	Total amount	Summation test
	10	234	BRL 109,877,366	0.0456
	12	190	BRL 104,132,939	0.0433
	11	208	BRL 87,720,460	0.0364
	17	114	BRL 85,948,151	0.0357
	68	37	BRL 80,584,943	0.0335
	15	162	BRL 70,752,611	0.0294
	16	139	BRL 67,394,183	0.0280
	14	166	BRL 66,918,817	0.0278
	25	110	BRL 66,881,381	0.0278
	38	64	BRL 66,550,185	0.0276

**Source:** (created by authors, 2020)

Finally, given the digits highlighted on Table 4, we select municipalities in which test variable leading digits begin with. The last line of the script is used to save the list of municipalities into a csv file for additional analysis. In practical terms, in case of auditing or fraud detection, this work suggests start by the chosen municipalities. As additional analysis we suggest include municipalities population to this file and compare the number of beneficiaries in suspected municipalities with the population. In case of Brazil this information is provided by IBGE – Geography and Statistics Brazilian Institute.

## Declaration of Competing Interest

The authors declare that they have no known competing financial interests or personal relationships that could have appeared to influence the work reported in this paper.
